# Supplementary material for: Acceptance and compliance with micronutrient powder (MNP) among children aged 6–23 months in northern Nigeria
Source: PLOS Glob Public Health. 2022 Oct 17;2(10):e0000961. doi: 10.1371/journal.pgph.0000961 (PMC10022258; doi:10.1371/journal.pgph.0000961)
Supplement: S1 Dataset — (ZIP) [file pgph.0000961.s008.zip › S8_Dataset/Spot Checks.zip!/Spot Checks/13072015_A_GU_2_SCc.pdf]

SPOT CHECK RECORDING FORM

State: Adamawa _ song__________ Community: lbolki_

Name of caregiver (female):……..zainab ahmadu…….

Name of caregiver (male): ………ahmadu musa…….…………………._

Sex of index child: female__ Age of index child (in months): 6month________

Spot Check Information

1. **Date of MNP distribution to household** (circle option below based on LGA name above)**:**
   - **Kebbi:** BirninKebbi&Jega LGA: **June 16**
   - **Kebbi:** Danko-wasagu: **June 17**
   - **Adamawa:**Song, Ganye: **June 18**
   - **Adamawa:**Guyuk **June 19**
2. **Date of Spot Check Observation: ___14/07/2015_____**
3. **Number of sachets remaining**(Ask the caregiver to show you and count the specific number of sachets remaining)**:**

   35

4. **Number of sachets that <u>should remain</u>using one MNP sachet per day** (Analyst only)**:**

   35

5. **Number of sachets remaining / number of sachets should be remaining**(Analyst only)**:**

   ___35/35______ = ____100_____ %

Note

The mother said she was given MNP on the 17th of June

Probes

Question 1. You have **XX** MNP sachets remaining. Explain how easy or difficult it was to use the MNP product on a daily basis for your child 6 – 23 months? (Circle one answer)

Very Difficult **Difficult** **Easy** **Very Easy**

Question 2. Describe any specific challenges that you faced that made the MNP difficult to use on a daily basis?

Some days my child rejects the kunu mixed with the product so what I do is to force her to eat it. That is the only difficulties I encountered. But other days .she agrees to take the kunu without any problem

Question 3
Pouring the MNP content into kunu and mixing it until is blends weel is what I in joy the most and my child does not throw up afterI feed her with the product.
Question 4. What questions do you have about the MNP based on your experience using it?

I want **to** know if it is the product that makes my child to have a black diarrhea .after using this product for a weeks so, I start monitoring that from child
Question 5. What specific recommendations do you have that we can do as a program to make the MNP easier to use on a daily basis?

I would like the federal ministry of health to bring a product to unseal daily and a lager packet.my recommendation are just these two I have mention another thing is fine.
